# Supplementary material for: Tuning the apparent hydrogen binding energy to achieve high-performance Ni-based hydrogen oxidation reaction catalyst
Source: Nat Commun. 2024 Feb 7;15:1137. doi: 10.1038/s41467-024-45370-4 (PMC10850486; doi:10.1038/s41467-024-45370-4)
Supplement: Supplementary file 1 — Supplementary Information [file 41467_2024_45370_MOESM1_ESM.pdf]

Supplementary information

Tuning the Apparent Hydrogen Binding Energy to Achieve High-Performance Ni-based Hydrogen Oxidation Reaction Catalyst

Xingdong Wang[1,2,†], Xuerui Liu[1,†], Jinjie Fang[1,†], Houpeng Wang[2], Xianwei Liu[1], Haiyong Wang[1], Chengjin Chen[1], Yongsheng Wang[1], Xuejiang Zhang[1], Wei Zhu[1] and Zhongbin Zhuang[1,3,*]

[1]State Key Lab of Organic-Inorganic Composites and Beijing Advanced Innovation Center for Soft Matter Science and Engineering, Beijing University of Chemical Technology, China.

[2]Research Institute of Petroleum Processing, SINOPEC, China.

[3]Beijing Key Laboratory of Energy Environmental Catalysis, Beijing University of Chemical Technology, China.

[†]These authors contributed equally: Xingdong Wang, Xuerui Liu and Jinjie Fang.

Supplementary Note 1. Calculation of the kinetic current and the exchange current density

The $j_{m@50mV}$ was the mass activity of electrode at η=50 mV, which was j_k normalized by the metal loadings. The j_k is the geometric area normalized value of i_k. The i_k was calculated by the Koutecky-Levich equation,

$$\frac{1}{i}=\frac{1}{i_k}+\frac{1}{i_d}$$

Where i is the measured current at a potential, i_k is the calculated kinetic current, i_d is the diffusion current. The i_d can be calculated by the Nernstian diffusion equation.

$$\eta_d=-RT/2F\ln(1-\frac{i_d}{i_l})$$

Where η_d is the over-potential of the electrode, R is the ideal gas constant (8.314 J·mol^{-1}·K^{-1}), T is the temperature (in Kelvin). F is the Faraday constant (96485 C mol^{-1}), i_l is the diffusion limiting current, which can be described by Levich equation.

$$i_l=0.62nFAD^{2/3}v^{-1/6}c_0\omega^{1/2}$$

Where n is the number of transferred electrons of the reaction, A is the geometric area of the electrode, D is the H_2 diffusion constant in the electrolyte, c_0 is the concentration of H_2, ω is the rotation speed of the electrode, and v is the kinetic viscosity.

The j_0 was defined as the exchange current density normalized to electrochemical active surface area (ECSA). The exchange current density was calculated by fitting the Butler-Volmer equation.

$$j_k=j_0(e^{\frac{\alpha F\eta}{RT}}-e^{\frac{-\beta F\eta}{RT}})$$

Where j_k is the kinetic current density of the electrode, η is the over-potential of the electrode. α and β is the transfer coefficient of anode and cathode, respectively. And $\alpha+\beta$=1 was set in the calculation.

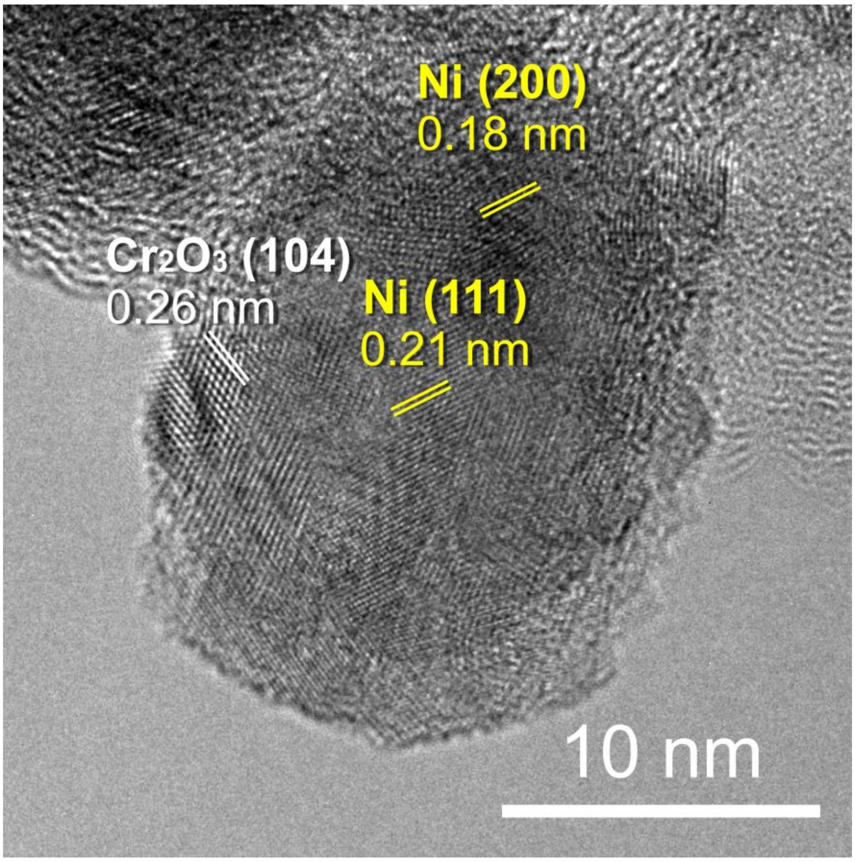

Supplementary Figure 1. HRTEM image of NiCuCr/C.

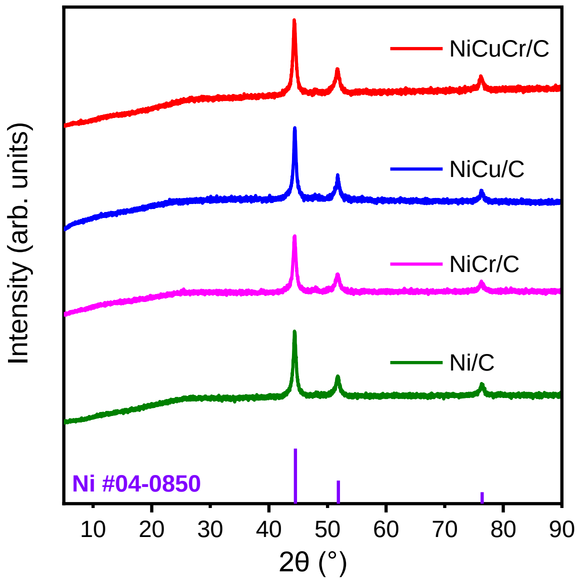

Supplementary Figure 2. XRD patterns of the Ni-based catalysts.

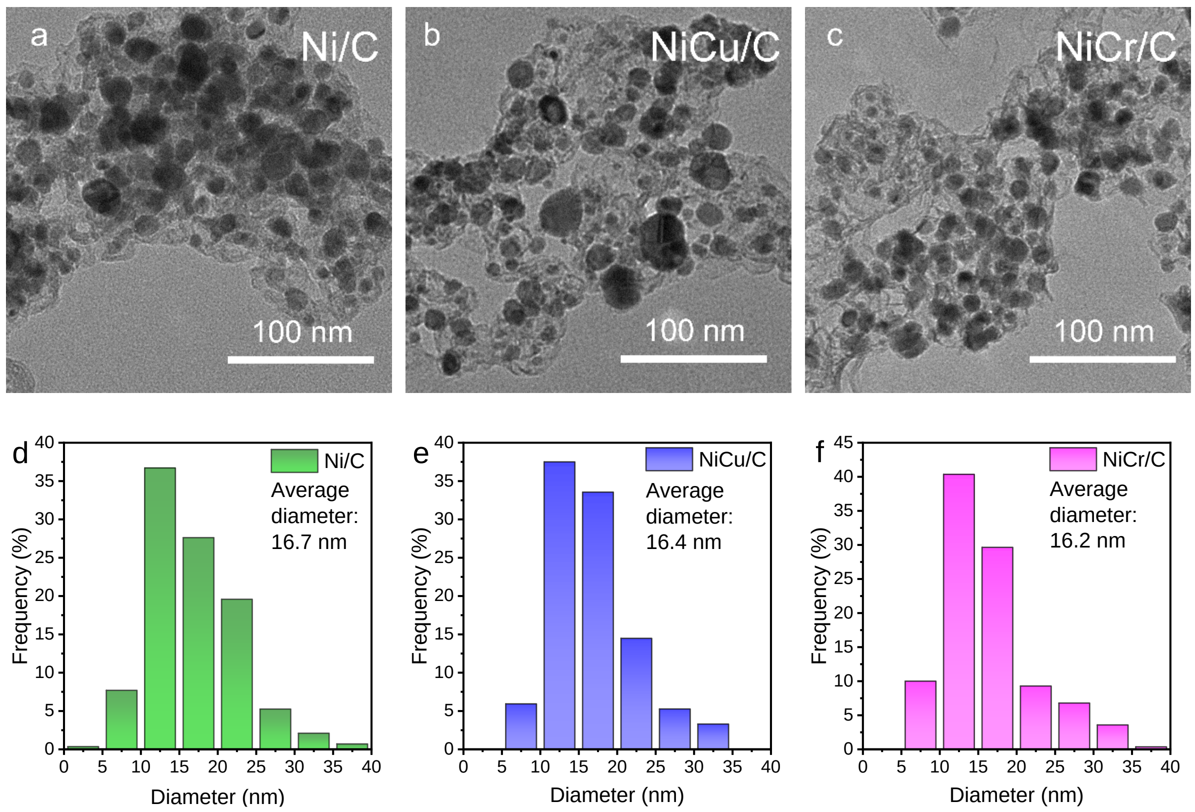

Supplementary Figure 3. TEM image of (a) Ni/C, (b) NiCu/C and (c) NiCr/C. Particle diameter distribution of (d) Ni/C, (e) NiCu/C and (f) NiCr/C. At least 100 particles were measured in statistics analysis.

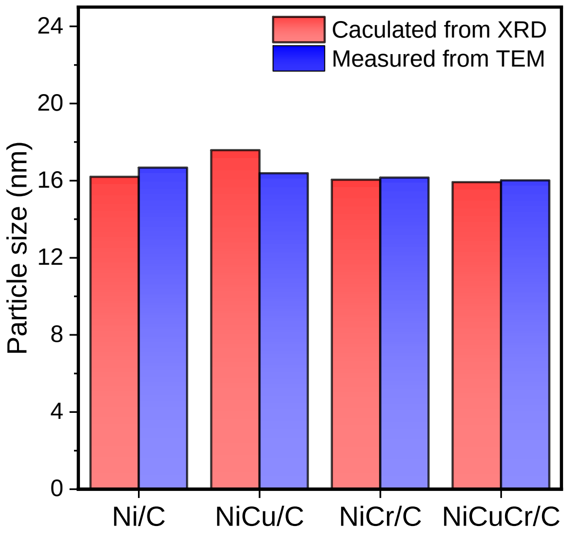

Supplementary Figure 4. The average diameter of Ni-based catalyst calculated from the XRD pattern and TEM image.

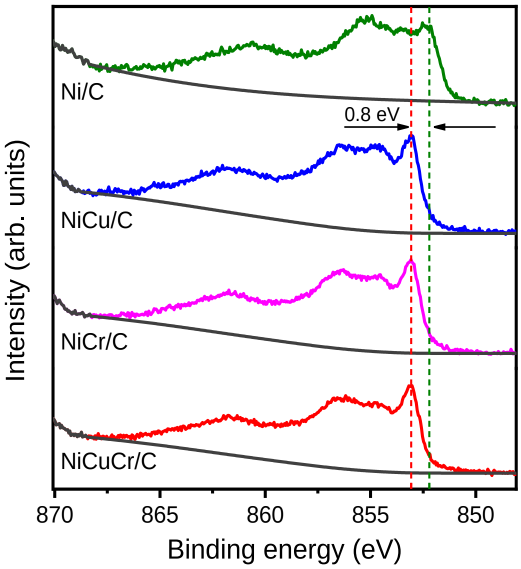

Supplementary Figure 5. High-resolution Ni 2*p* XPS spectra of Ni/C, NiCu/C, NiCr/C and NiCuCr/C.

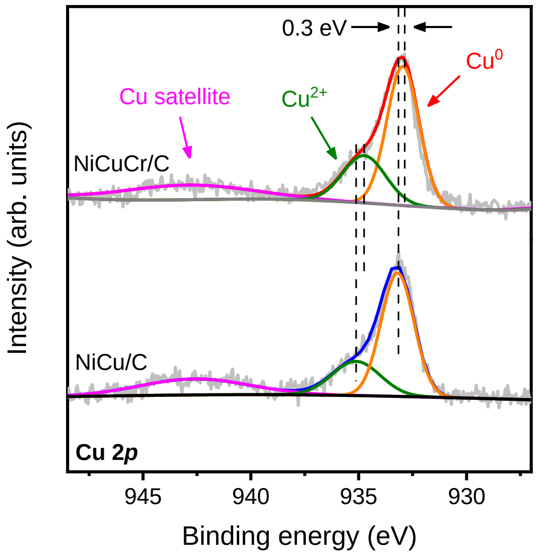

Supplementary Figure 6. High resolution Cu 2*p* XPS spectra of NiCuCr/C and NiCu/C.

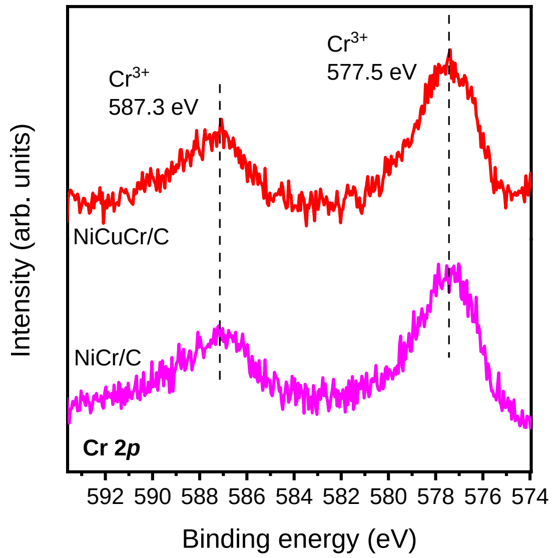

Supplementary Figure 7. High resolution Cr 2*p* XPS spectra of NiCuCr/C and NiCr/C.

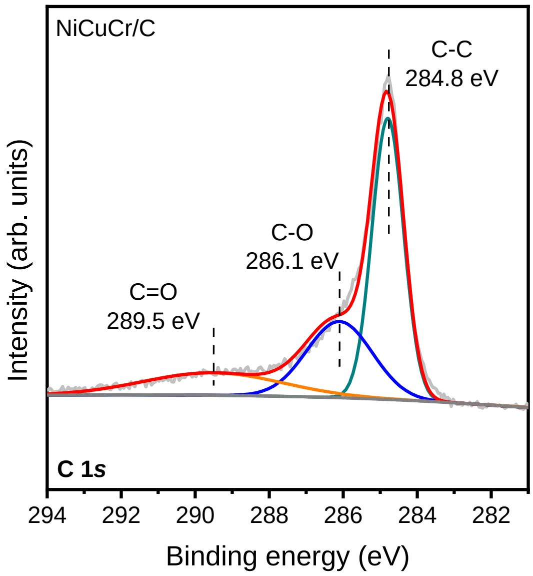

Supplementary Figure 8. High resolution C 1*s* XPS spectra of NiCuCr/C.

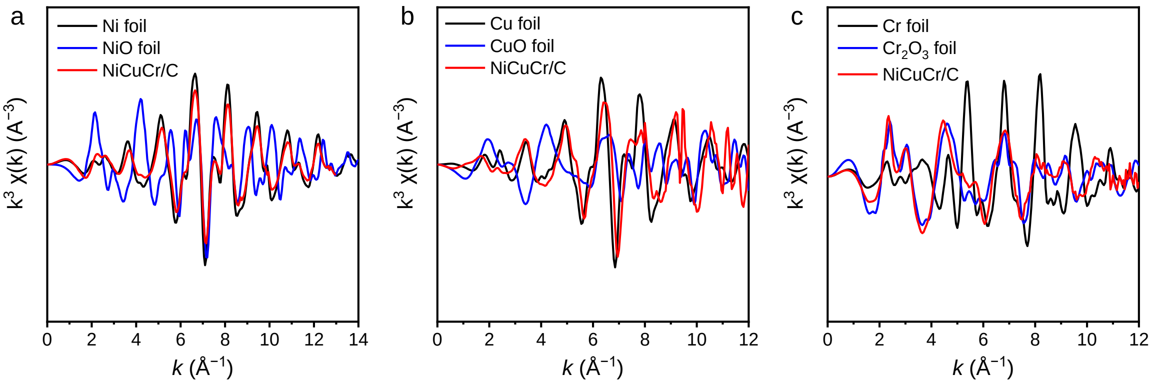

Supplementary Figure 9. (a) Ni, (b) Cu and (c) Cr K-edge EXAFS oscillation spectra of respective metal foil, typical metal oxide and NiCuCr/C.

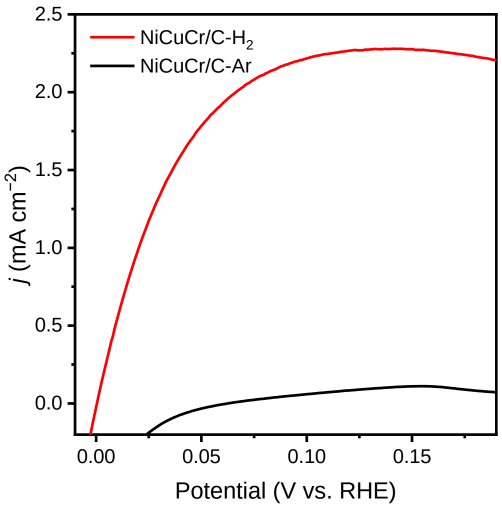

Supplementary Figure 10. The polarization curves of NiCuCr/C in H_2 and Ar-saturated 0.1 M KOH electrolyte at a scan rate of 1 mV s^{-1}

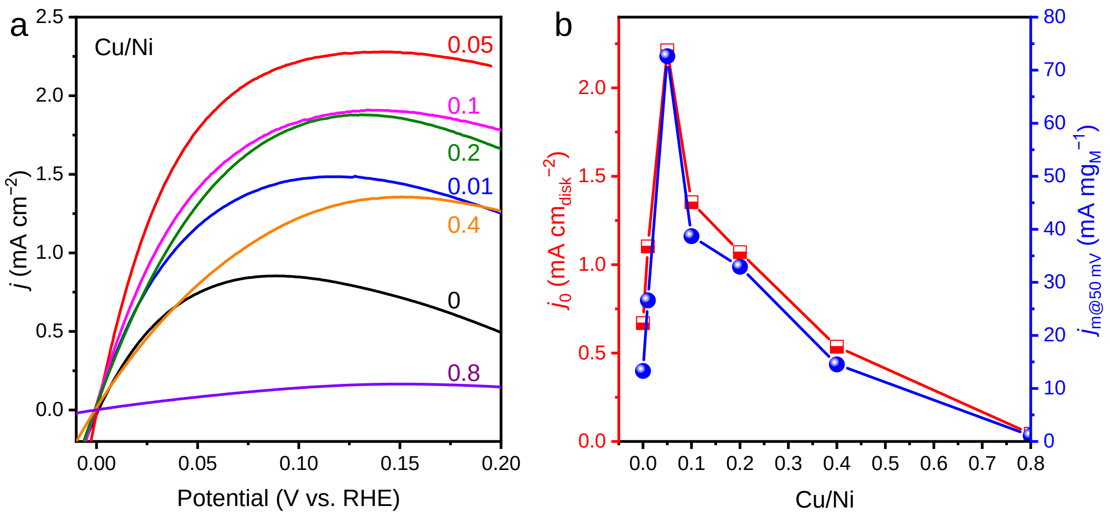

Supplementary Figure 11. The HOR performance of NiCuCr with different Cu amounts. (a) Polarization curves of NiCuCr/C catalyst with different Cu/Ni ratios in H_2-satureated 0.1 M KOH. The catalyst loading was 0.080 mg_M cm^{-2} for catalysts. The rotating speed was 1600 rpm and the scan rate was 1 mV s^{-1}. (b) Exchange current density and mass activity (@50 mV) of NiCuCr/C catalyst with different Cu/Ni ratios.

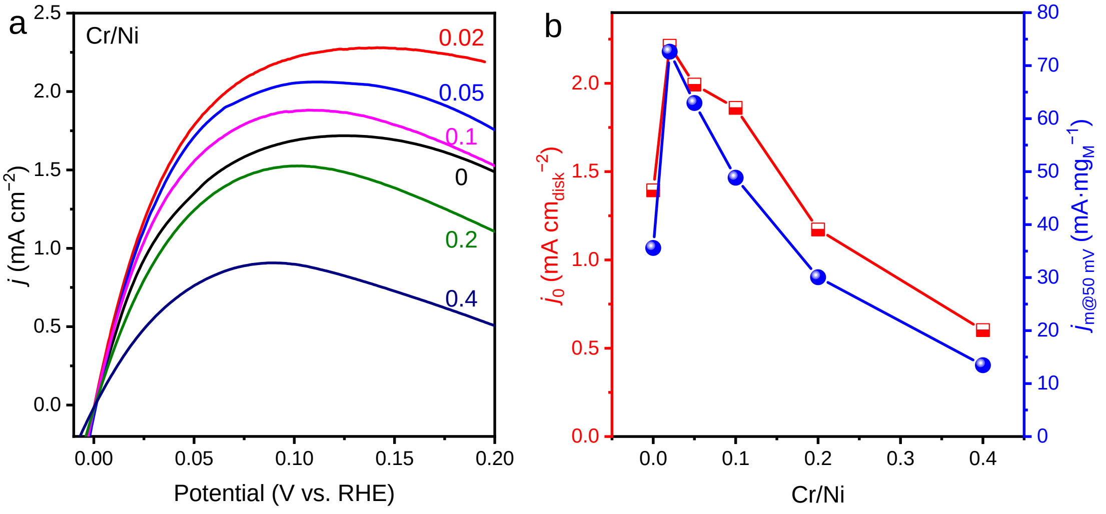

Supplementary Figure 12. The HOR performance of NiCuCr with different Cr amounts. (a) Polarization curves of NiCuCr/C catalyst with different Cr/Ni ratios in H_2-satureated 0.1 M KOH. The catalyst loading was 0.080 mg_M cm^{-2} for catalysts. The rotating speed was 1600 rpm and the scan rate was 1 mV s^{-1}. (b) Exchange current density and mass activity (@50 mV) of NiCuCr/C catalyst with different Cr/Ni ratios.

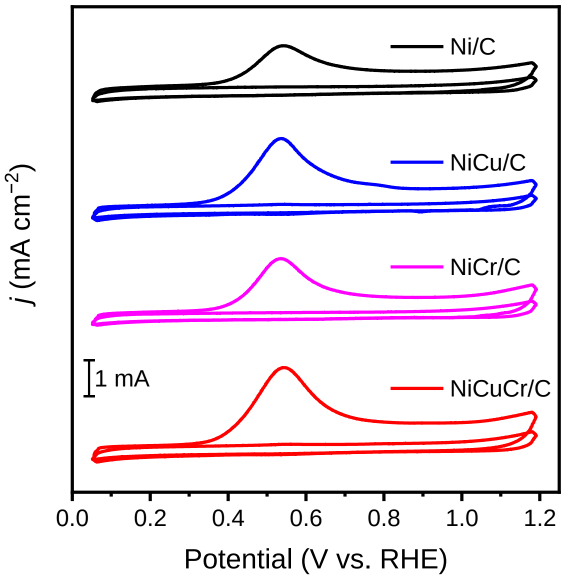

Supplementary Figure 13. CO stripping curves of Ni-based catalysts in 0.1 M KOH, the scan rate was 20 mV s^{-1} for all catalysts.

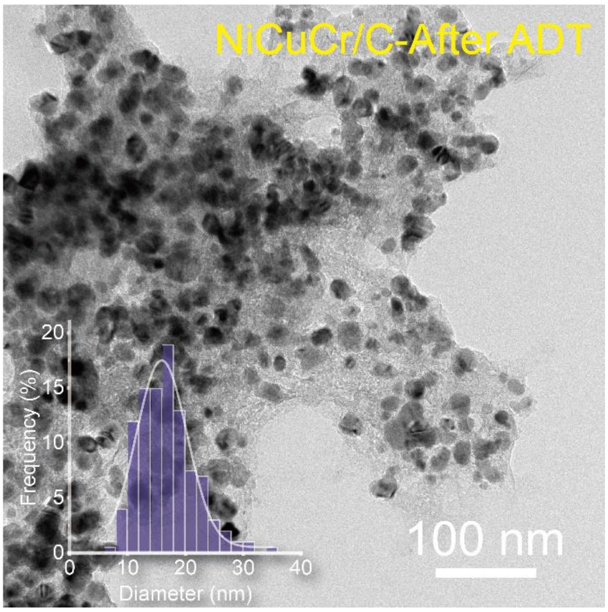

Supplementary Figure 14. TEM image of NiCuCr/C after ADT. The insert is the statistical histogram of the size of the particles.

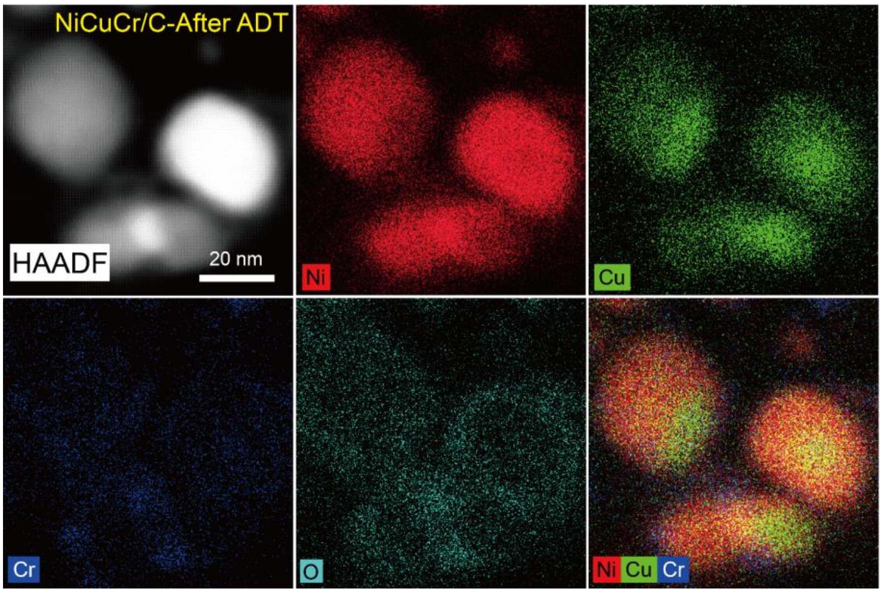

Supplementary Figure 15. HAADF-STEM elemental mapping image of NiCuCr/C after ADT.

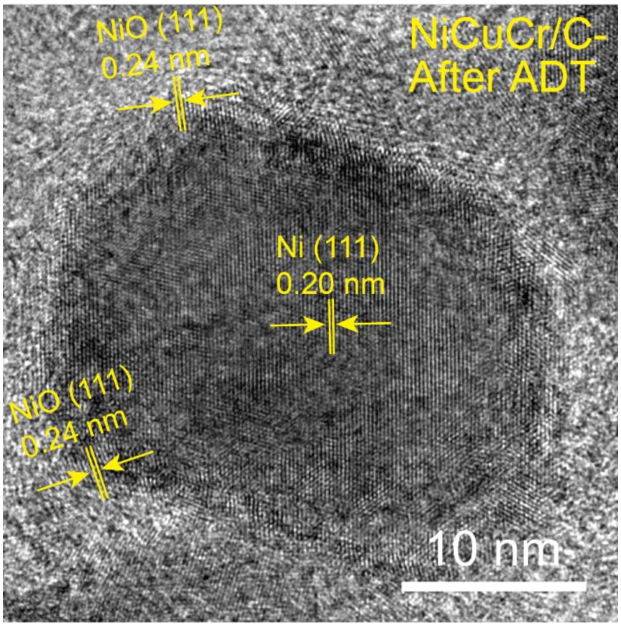

Supplementary Figure 16. HRTEM image of NiCuCr/C after ADT.

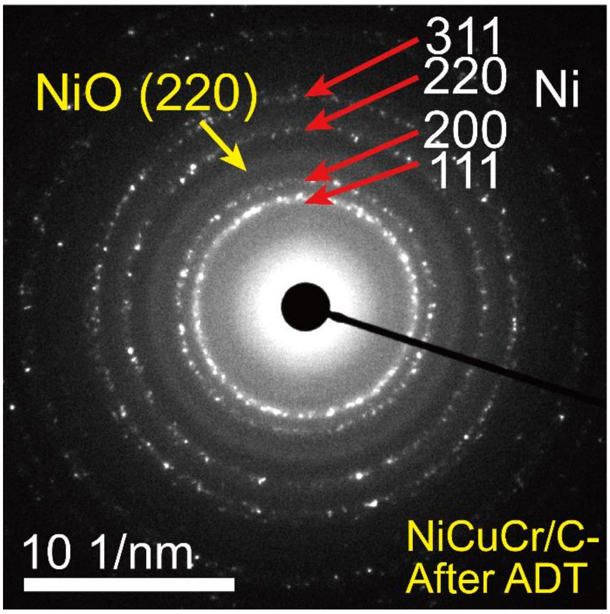

Supplementary Figure 17. SAED pattern of NiCuCr/C after ADT.

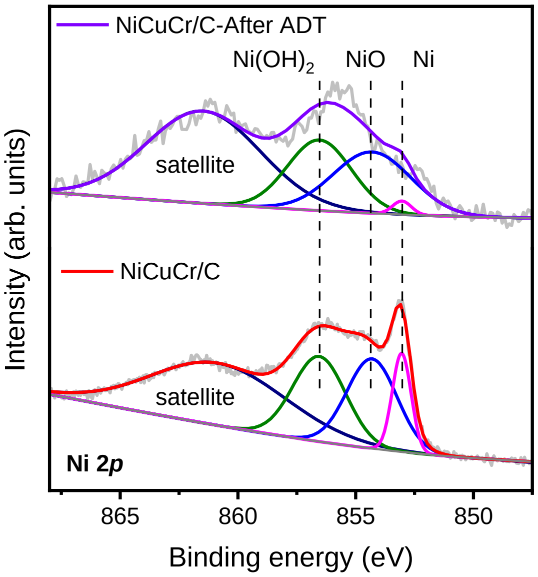

Supplementary Figure 18. High resolution Ni 2*p* spectra of NiCuCr/C after ADT and NiCuCr/C.

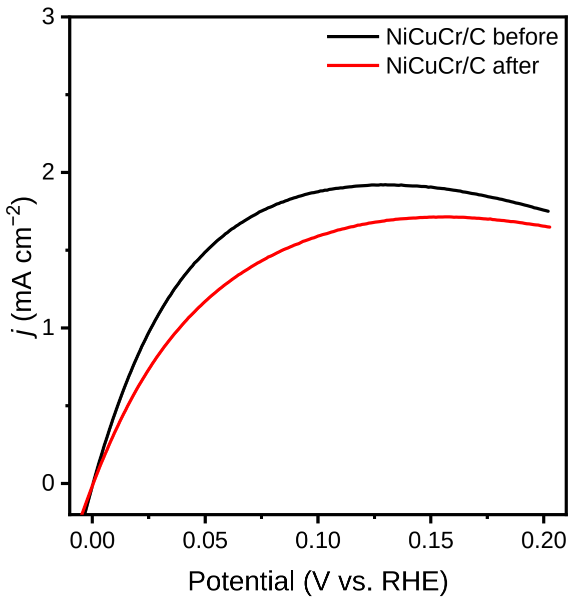

Supplementary Figure 19. Polarization curves of NiCuCr/C in H_2-saturated 0.1 M KOH electrolyte before and after 100 ppm CO poisoning test. The scan rate was 1 mV s^{-1}.

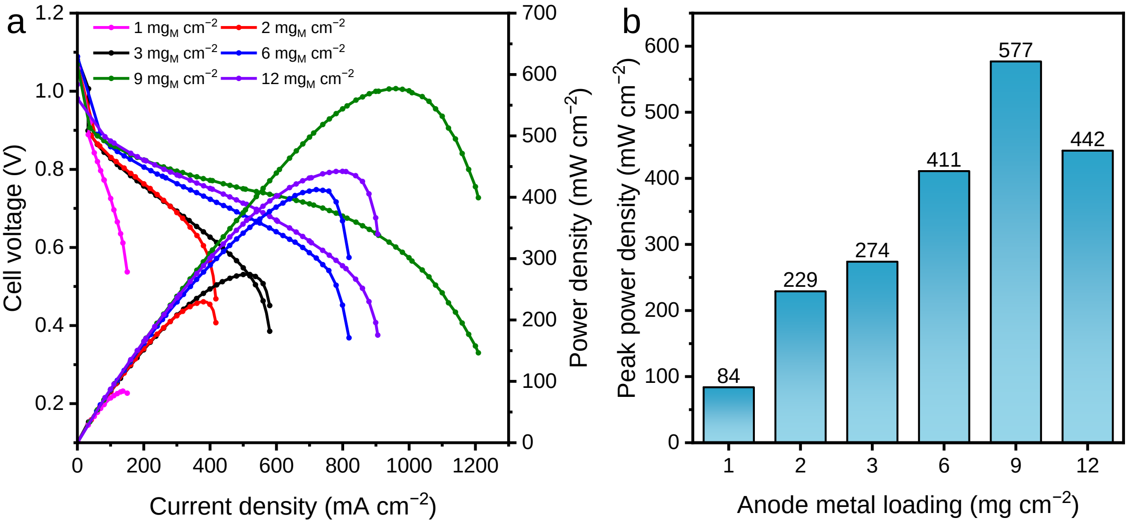

Supplementary Figure 20. (a) Polarization and power density curves of NiCuCr/C catalyst in 1, 2, 3, 6, 9 and 12 mg_M cm^{-2} catalyst loading. (b) Corresponding peak power densities of NiCuCr/C HEMFCs using different catalyst loadings.

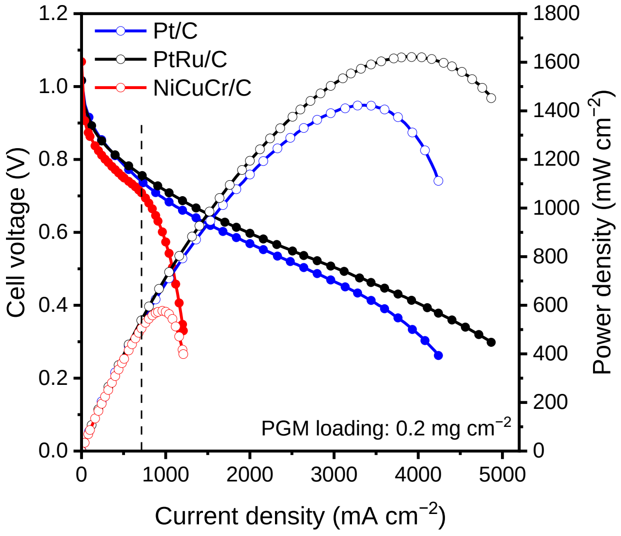

Supplementary Figure 21. H_2-O_2 HEMFC polarization and power density curves with Pt/C, PtRu/C or NiCuCr/C anode. The anode catalyst loading was 9 $mg_{n\text{-}PGM}$ cm^{-2} and 0.2 mg_{PGM} cm^{-2}, respectively. And the cathode catalyst loading was 0.2 mg_{Pt} cm^{-2} using commercial 40% Pt/C. The anode, cathode humidifier temperatures, and the cell temperature were 78, 80, and 80 °C, respectively. The back pressure was 2.0 bar, and the flow rates of H_2 and O_2 were both 1000 sccm.

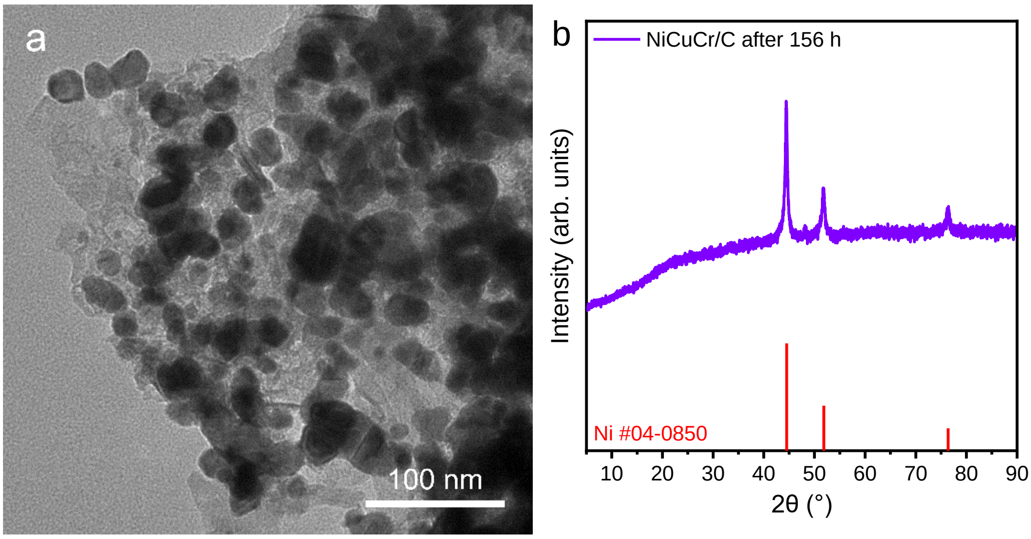

Supplementary Figure 22. The NiCuCr/C after 156 h of HEMFC test. (a) TEM image. (b) XRD pattern.

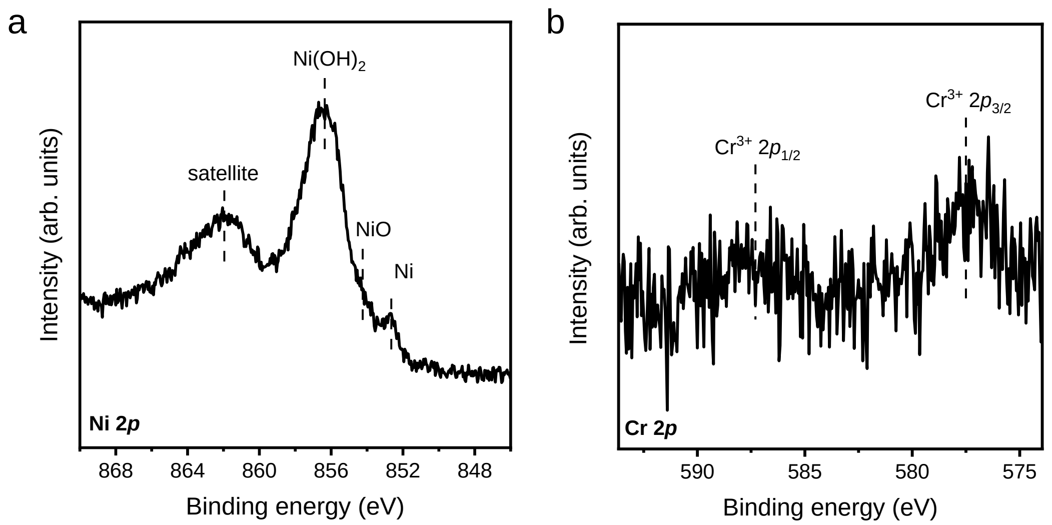

Supplementary Figure 23. High resolution (a) Ni 2*p* and (b) Cr 2*p* XPS spectra of NiCuCr/C after 156 h of HEMFC test.

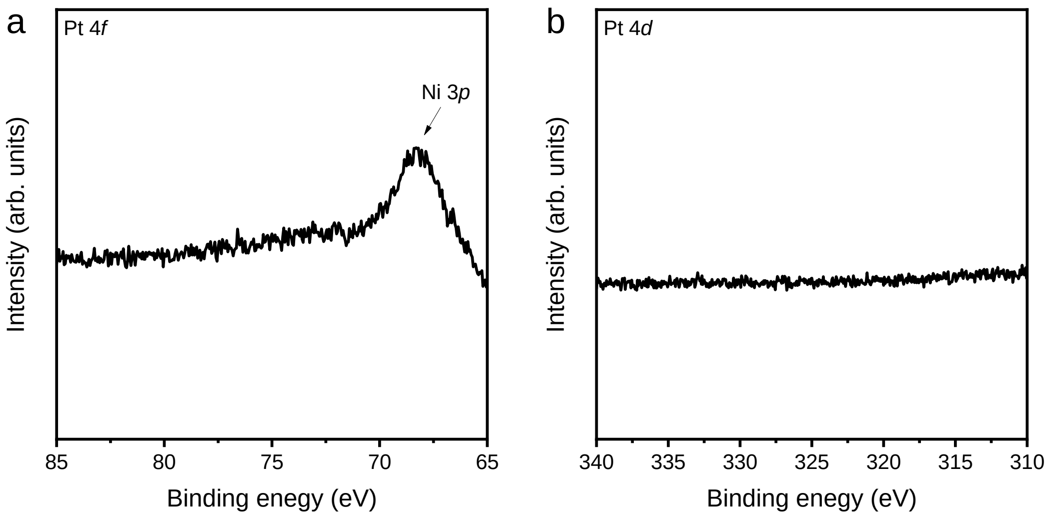

Supplementary Figure 24. XPS result to exclude the possibility of Pt migration from cathode to anode. (a) Pt 4*f* spectra. (b) Pt 4*d* spectra.

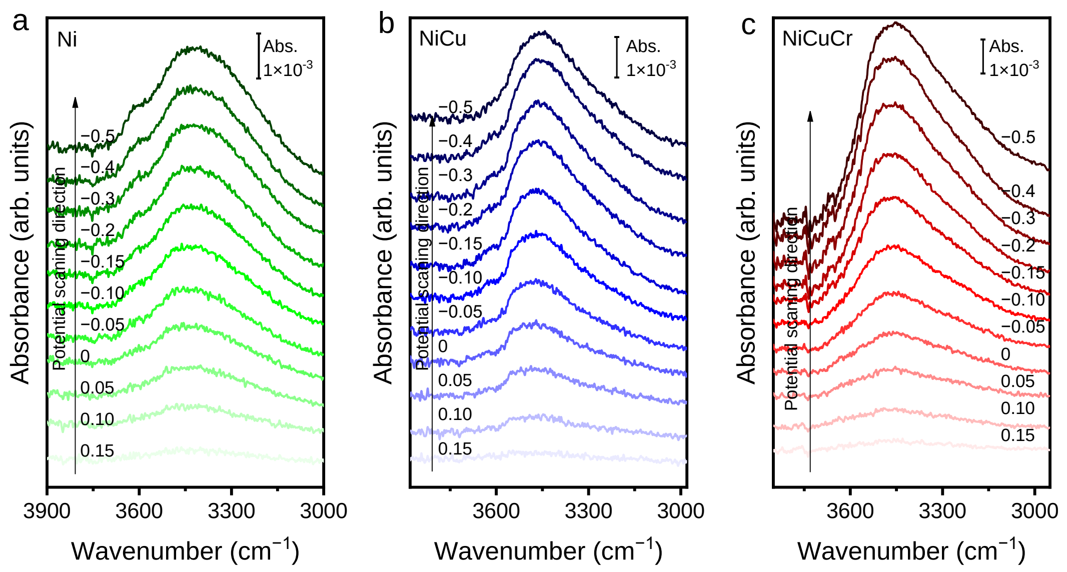

Supplementary Figure 25. The potential-dependent OH stretching features of in situ SEIRAS spectra of HER/HOR measured from 0.15 to −0.5 V (vs. RHE) of (a) Ni, (b) NiCu and (c) NiCuCr.

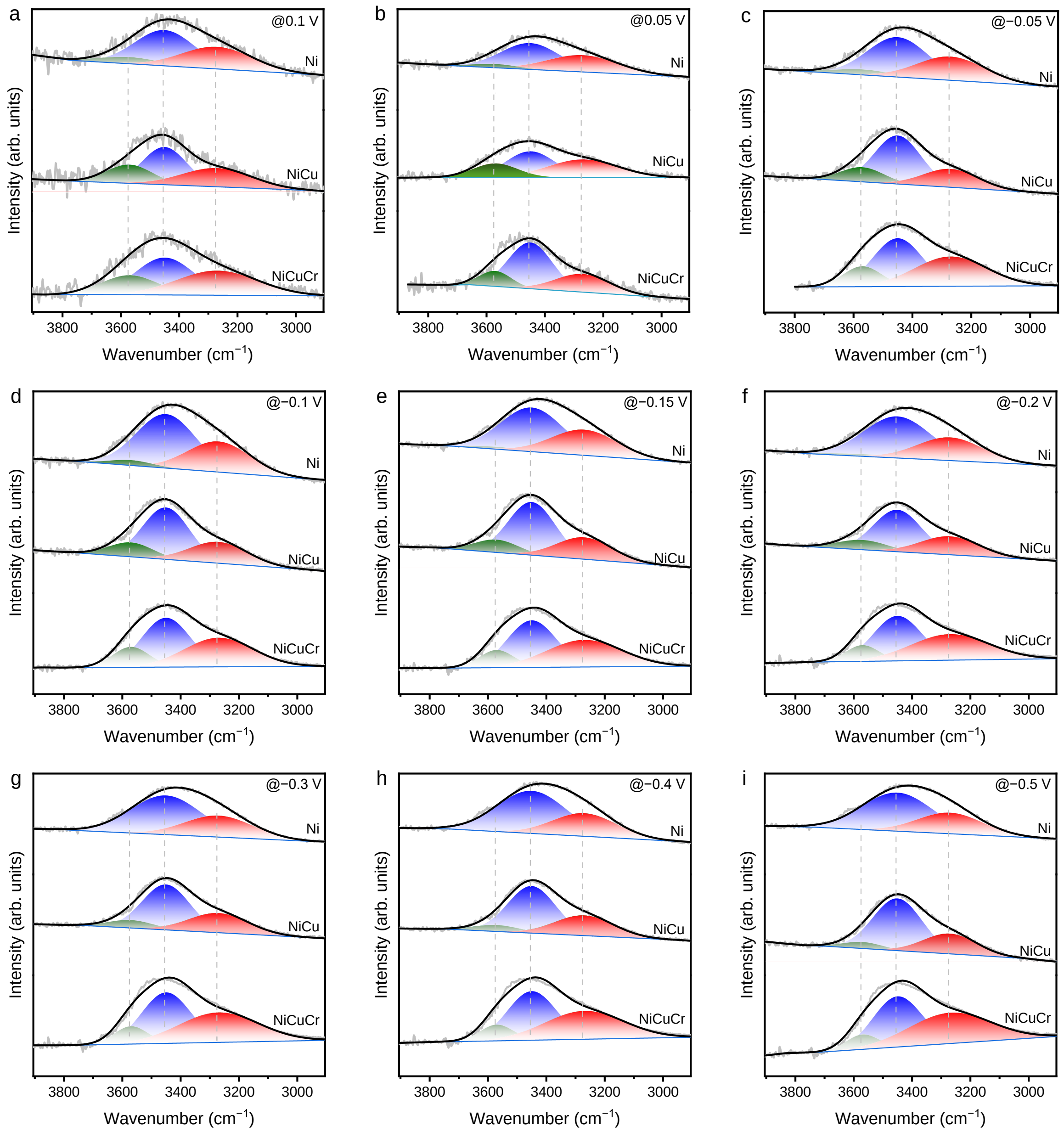

Supplementary Figure 26. Deconvolution of OH stretching features of in situ SEIRAS spectra of HOR/HER on Ni, NiCu and NiCuCr at (a) 0.1 V. (b) 0.05 V. (c) −0.05 V. (d) −0.1V. (e) −0.15 V. (f) −0.2 V. (g) −0.3 V. (h) −0.4 V and (i) −0.5 V.

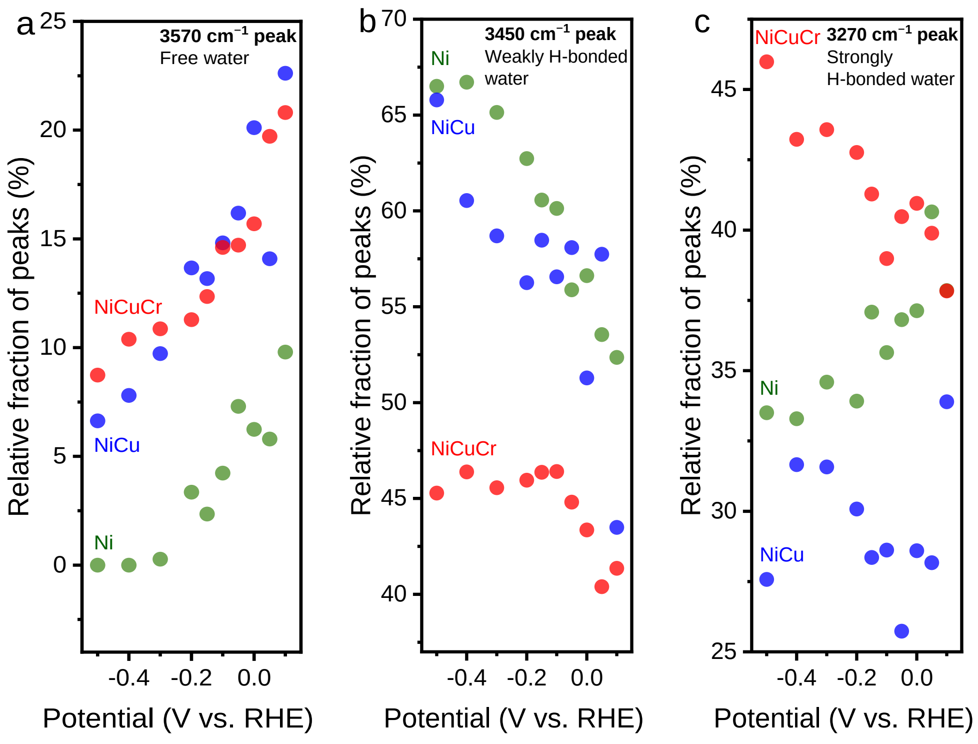

Supplementary Figure 27. The potential dependence of the relative fractions of (a) free water, (b) weakly H-bonded water, and (c) strongly H-bonded water according to Supplementary Figure 26.

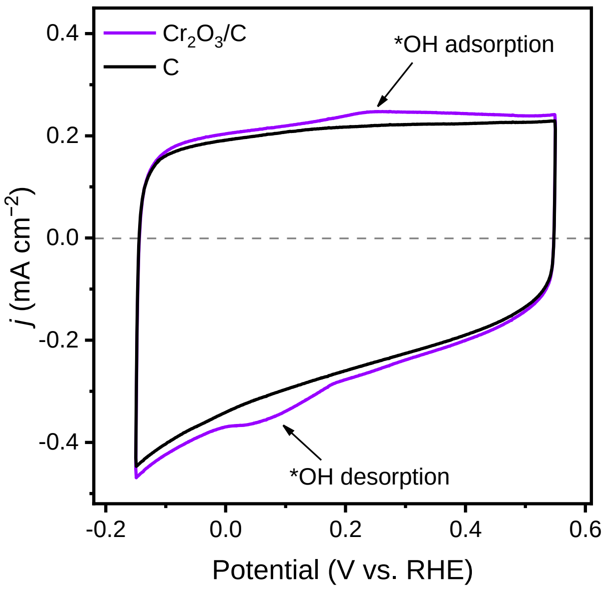

Supplementary Figure 28. The CV curves of Cr_2O_3/C and C in 0.1 M KOH, the scan rate was 50 mV s^{-1}. The Cr_2O_3/C was synthesized using the impregnation and calcination method.

Supplementary Table 1. ICP-OES analysis results of weight ratio of Ni/C, NiCu/C, NiCr/C and NiCuCr/C.

Catalyst	Ni (%)	Cu (%)	Cr (%)
Ni/C	61.5	-	-
NiCu/C	59.1	4.0	-
NiCr/C	61.0	-	1.1
NiCuCr/C	57.6	3.9	1.1

Supplementary Table 2. Comparison of the HOR activity between NiCuCr/C and the reported Ni-based catalysts for HOR in 0.1 M KOH or NaOH.

Catalyst	Electrolyte	$j_{0,m}$ ($A \cdot g_M^{-1}$)	j_0 ($\mu A \cdot cm_M^{-2}$)	$j_{m@50mV}$ ($A \cdot g_M^{-1}$)	$j_{k@50mV}$ ($\mu A \cdot cm_M^{-2}$)	Ref.
**NiCuCr/C**	**0.1 M KOH**	**27.7**	**135.5**	**72.6**	**354.9**	**This work**
Ni-H_2-NH_3	0.1 M KOH	-	70	59.2	-	1
Ch-activated Ni_{ED}/XC-72	0.1 M NaOH	22.4 ± 4.3	56 ± 10	32.1 ± 4.8	83 ± 12	2
Ni_3@(h-BN)$_1$/C-700NH_3	0.1 M NaOH	-	23	-	-	3
Ni-H_2-2%	-	24.41	28.0	50.4	-	4
np-Ni_3N	-	10.3	-	29.75	-	5
Ni/NiO/C700	-	-	26	5.0	-	6
Ni_3N/C	0.1 M KOH	12.0	14	24.38	-	7
Ni/SC	0.1 M KOH	7.44 ± 0.88	40.2 ± 2.8	11.0	-	8
CeO_2(r)Ni/C-1	0.1 M KOH	-	38	12.28	-	9
Ni/N-CNT	0.1 M KOH	-	28	9.3	-	10
Ni@C-500 °C	0.1 M KOH	-	32	-	-	11
CoNiMo	0.1 M KOH	-	15 ± 2	-	44 ± 5	12
Ni/V_2O_3	0.1 M KOH	-	38	42.1	-	13
Ni/Ni_3N-C	0.1 M KOH	5.186	36.45	12.176	-	14
Ni-400 ($NiO_{0.41}$)	0.1 M NaOH	-	18	-	140	15

Supplementary Table 2. (Continued)

Catalyst	Electrolyte	$j_{0,m}$ ($A·g_M^{-1}$)	j_0 ($\mu A·cm_M^{-2}$)	$j_{m@50mV}$ ($A·g_M^{-1}$)	$j_{k@50mV}$ ($\mu A·cm_M^{-2}$)	Ref.
Ni@NC/PEI-XC	0.1 M KOH	7.84	38	24.4	-	16
Ni/TN-CNS-0.3	0.1 M KOH	-	30	-	-	17
NiO−Ni/CNT	0.1 M KOH	3.715	359 ± 9	-	-	18
Ni@O_i-Ni	0.1 M KOH	-	72	85.63	-	19
4.3%N–Ni	0.1 M KOH	-	41	77.13	-	20
Ni_3N-r	0.1 M KOH	-	38	40.69	-	21
NiCu/KB	0.1 M KOH	0.9 ± 0.1	25 ± 1	-	-	22
hcp/fcc-Ni-C	0.1 M KOH	-	30.88 ± 1.07	12.28 ± 1.57		23
NiMo/N-MSUF-C	0.1 M KOH	2.1	-	-	-	24
60% Ni/KB-600	0.1 M NaOH	7.02 ± 0.1	28.6 ± 0.3	-	-	25

Supplementary Table 3. Comparison of the H_2-O_2 HEMFC performances between NiCuCr/C and the reported Ni-based anode.

Anode catalyst	Cathode catalyst	Cell temperature (°C)	Backpressure (anode/cathode, kPa)	Peak power density (mW cm^{-2})	Stability	Ref.
**NiCuCr/C**	**Pt/C**	**80**	**200/200**	**577**	**156 h@0.7 V**	**This work**
Ni@CN_x	Pt/C	80	200/200	480	100 h@200 mA cm^{-2}	[26]
Co-$MoNi_4$	Pt/C	95	200/200	525	-	[27]
$Ni_{52}Mo_{13}Nb_{35}$	Pt/C	90	200/200	390	50 h@200 mA cm^{-2}	[28]
Ni_3N	Pt/C	90	200/200	532	25 h@100 mA cm^{-2}	[29]
Ni@O_i-Ni	Pt/C	80	200/200	274	-	[19]
Ni-H_2-NH_3	Pt/C	95	250/250	628	40 h@0.7 V	[1]
Ni/Ni_3N-C	Pt/C	80	200/200	222.5	-	[14]
Mo-decorated Ni_3N	Pt/C	70	100/100	180	30 h@0.6 V	[30]
Ni@NC/PEI-XC	Pt/C	80	200/200	241	-	[16]
Ni@C-500°C	Pt/C	80	200/200	160	120 h@0.7 V	[11]
NiCu/KB	Pd/C	80	138/138	350	-	[22]
NiMo/KB	Pd/C	70	138/138	120	115 h@0.7 V	[31]

Supplementary Table 4. Comparison of the peak power density of H_2-O_2 HEMFCs operated with Ni-based anode and PGM-free cathode.

Anode catalyst	Cathode catalyst	Cell temperature (°C)	Backpressure (anode/cathode, kPa)	Flow rate (anode/cathode, sccm)	Peak power density (mW cm^{-2})	Ref.
**NiCuCr/C**	**Ag/C**	**80**	**200/200**	**1000/1000**	**335**	**This work**
Ni@CN_x	$MnCo_2O_4$	80	200/200	500/500	210	[26]
Ni-H_2-NH_3	$MnCo_2O_4$/C	95	250/250	200/200	488	[1]
Ni_3N	ZrN	90	200/200	800/1600	256	[29]
Ni_7Fe	N-doped carbon	95	200/0	10/200	56	[32]
NiCo/C	Co_3O_4	60	130/130	1000/500	22	[33]
Ni-W	CoPPY/C	60	0/0	50/50	40	[34]
Ni/C	Ag/C	80	250/250	200/200	76	[35]
NiCr	Ag/C	60	130/130	-	50	[36]

Reference

1. Ni W, *et al.* An Efficient Nickel Hydrogen Oxidation Catalyst for Hydroxide Exchange Membrane Fuel Cells. *Nat Mater* **21**, 804-810 (2022).
2. Oshchepkov AG, *et al.* Nanostructured Nickel Nanoparticles Supported on Vulcan Carbon as a Highly Active Catalyst for the Hydrogen Oxidation Reaction in Alkaline Media. *J Power Sources* **402**, 447-452 (2018).
3. Gao LJ, *et al.* A Nickel Nanocatalyst Within a h-BN Shell for Enhanced Hydrogen Oxidation Reactions. *Chem sci* **8**, 5728-5734 (2017).
4. Ni WY, Wang T, Schouwink PA, Chuang YC, Chen HM, Hu XL. Efficient Hydrogen Oxidation Catalyzed by Strain-Engineered Nickel Nanoparticles. *Angew Chem Int Ed* **59**, 10797-10801 (2020).
5. Wang T, *et al.* Weakening Hydrogen Adsorption on Nickel *via* Interstitial Nitrogen Doping Promotes Bifunctional Hydrogen Electrocatalysis in Alkaline Solution. *Energy Environ Sci* **12**, 3522-3529 (2019).
6. Yang Y, *et al.* Enhanced Electrocatalytic Hydrogen Oxidation on Ni/NiO/C Derived from a Nickel-Based Metal-Organic Framework. *Angew Chem Int Ed* **58**, 10644-10649 (2019).
7. Ni WY, Krammer A, Hsu CS, Chen HM, Schuler A, Hu XL. Ni_3N as an Active Hydrogen Oxidation Reaction Catalyst in Alkaline Medium. *Angew Chem Int Ed* **58**, 7445-7449 (2019).
8. Yang FL, Bao X, Zhao YM, Wang XW, Cheng GZ, Luo W. Enhanced HOR Catalytic Activity of PGM-free Catalysts in Alkaline Media: the Electronic Effect Induced by Different Heteroatom Doped Carbon Supports. *J Mater Chem A* **7**, 10936-10941 (2019).
9. Yang F, *et al.* Boosting Hydrogen Oxidation Activity of Ni in Alkaline Media Through Oxygen-vacancy-rich CeO_2 /Ni Heterostructures. *Angew Chem Int Ed* **58**, 14179-14183 (2019).
10. Zhuang Z, *et al.* Nickel Supported on Nitrogen-doped Carbon Nanotubes as Hydrogen Oxidation Reaction Catalyst in Alkaline Electrolyte. *Nat Commun* **7**, 10141 (2016).
11. Gao YF, *et al.* Improving the Antioxidation Capability of the Ni Catalyst by Carbon Shell Coating for Alkaline Hydrogen Oxidation Reaction. *ACS Appl Mater Interfaces* **12**, 31575-31581 (2020).
12. Sheng WC, *et al.* Non-precious Metal Electrocatalysts with High Activity for Hydrogen Oxidation Reaction in Alkaline Electrolytes. *Energy Environ Sci* **7**, 1719-1724 (2014).
13. Duan Y, *et al.* Interfacial Engineering of Ni/V_2O_3 Heterostructure Catalyst for Boosting Hydrogen Oxidation Reaction in Alkaline Electrolytes. *Angew Chem Int Ed* **62**, e202217275 (2023).
14. Su L, Gong D, Yao N, Li Y, Li Z, Luo W. Modification of the Intermediate Binding Energies on Ni/Ni_3N Heterostructure for Enhanced Alkaline Hydrogen Oxidation Reaction. *Adv Funct Mater* **31**, 2106156 (2021).
15. Sun C, Zhao P, Yang Y, Li Z, Sheng W. Lattice Oxygen-Induced d-Band Shifting for Enhanced Hydrogen Oxidation Reaction on Nickel. *ACS Catal* **12**, 11830-11837 (2022).
16. Wang J, *et al.* Ultrafine Nickel Nanoparticles Encapsulated in N-Doped Carbon Promoting Hydrogen Oxidation Reaction in Alkaline Media. *ACS Catal* **11**, 7422-7428 (2021).
17. Jiang S, *et al.* Ni Nanoparticles Supported on Carbon Nanosheets with Tunable N Doping Content for Hydrogen Oxidation Reaction. *Chem Phys Lett* **728**, 19-24 (2019).
18. Campos-Roldán CA, Calvillo L, Boaro M, de Guadalupe González-Huerta R, Granozzi G, Alonso-Vante N. NiO–Ni/CNT as an Efficient Hydrogen Electrode Catalyst for a Unitized Regenerative Alkaline Microfluidic Cell. *ACS Appl Energy Mater* **3**, 4746-4755 (2020).
19. Men Y, *et al.* Oxygen-Inserted Top-Surface Layers of Ni for Boosting Alkaline Hydrogen Oxidation Electrocatalysis. *J Am Chem Soc* **144**, 12661-12672 (2022).
20. Zhao X, *et al.* Nitrogen-inserted Nickel Nanosheets with Controlled Orbital Hybridization and Strain

Fields for Boosted Hydrogen Oxidation in Alkaline Electrolytes. *Energy Environ Sci* **15**, 1234-1242 (2022).

21. Zhao X, Li X, An L, Zheng L, Yang J, Wang D. Controlling the Valence-Electron Arrangement of Nickel Active Centers for Efficient Hydrogen Oxidation Electrocatalysis. *Angew Chem Int Ed* **61**, e202206588 (2022).
22. Roy A*, et al.* Nickel–copper Supported on a Carbon Black Hydrogen Oxidation Catalyst Integrated into an Anion-exchange Membrane Fuel Cell. *Sustain Energy Fuels* **2**, 2268-2275 (2018).
23. Li Y, Li Z, Cong H, Yang C, Cheng G, Luo W. Boosting Hydrogen Oxidation Performance of Phase-Engineered Ni Electrocatalyst under Alkaline Media. *ACS Sustainable Chem Eng* **10**, 3682-3689 (2022).
24. Park J*, et al.* Effect of Support for Non-Noble NiMo Electrocatalyst in Alkaline Hydrogen Oxidation. *Adv Sustainable Syst* **6**, 2100226 (2022).
25. Simonov PA*, et al.* Highly Active Carbon-supported Ni Catalyst Prepared by Nitrate Decomposition with a Sacrificial Agent for the Hydrogen Oxidation Reaction in Alkaline Medium. *J Electroanal Chem* **852**, 113551 (2019).
26. Gao Y*, et al.* A Completely Precious Metal-free Alkaline Fuel Cell with Enhanced Performance Using a Carbon-coated Nickel Anode. *Proc Natl Acad Sci USA* **119**, e2119883119 (2022).
27. Yang Y*, et al.* Suppressing Electron Back-donation for a Highly CO-tolerant Fuel Cell Anode Catalyst via Cobalt Modulation. *Angew Chem Int Ed* **61**, e202208040 (2022).
28. Gao F-Y*, et al.* Nickel–Molybdenum–Niobium Metallic Glass for Efficient Hydrogen Oxidation in Hydroxide Exchange Membrane Fuel Cells. *Nat Catal* **5**, 993-1005 (2022).
29. Zhang X-L, Hu S-J, Wang Y-H, Shi L, Yang Y, Gao M-R. Plasma-Assisted Synthesis of Metal Nitrides for an Efficient Platinum-Group-Metal-Free Anion-Exchange-Membrane Fuel Cell. *Nano Lett* **23**, 107-115 (2023).
30. Chen F*, et al.* Mo-Decorated Ni3N Nanostructures for Alkaline Polymer Electrolyte Fuel Cells. *ACS Appl Nano Mater* **4**, 11473-11479 (2021).
31. Kabir S*, et al.* Platinum Group Metal-free NiMo Hydrogen Oxidation Catalysts: High Performance and Durability in Alkaline Exchange Membrane Fuel Cells. *J Mater Chem A* **5**, 24433-24443 (2017).
32. Biemolt J*, et al.* An Anion-exchange Membrane Fuel Cell Containing Only Abundant and Affordable Materials. *Energy Technol-Ger* **9**, 2000909 (2021).
33. Men Truong V*, et al.* Platinum and Platinum Group Metal-Free Catalysts for Anion Exchange Membrane Fuel Cells. *Energies* **13**, 582 (2020).
34. Hu Q, Li G, Pan J, Tan L, Lu J, Zhuang L. Alkaline Polymer Electrolyte Fuel Cell with Ni-based Anode and Co-based Cathode. *Int J Hydrogen Energy* **38**, 16264-16268 (2013).
35. Gu S*, et al.* An Efficient Ag-ionomer Interface for Hydroxide Exchange Membrane Fuel Cells. *Chem Commun* **49**, 131-133 (2013).
36. Lu SF, Pan J, Huang AB, Zhuang L, Lu JT. Alkaline Polymer Electrolyte Fuel Cells Completely Free from Noble Metal Catalysts. *Proc Nat Acad Sci USA* **105**, 20611-20614 (2008).
